# Acceptance and commitment therapy improves the quality of life of breast cancer patients by reducing helplessness: a randomized controlled trial

**DOI:** 10.1186/s12904-026-02049-5

**Published:** 2026-03-05

**Authors:** Wenjun Song, Nurul Izzah Shari, Mohammad Farris Iman Leong Bin Abdullah, Hasmah Binti Hussin, Ruiling Zhang, Nor Shuhada Mansor

**Affiliations:** 1 The Second Affiliated Hospital of Zunyi Medical University, Zunyi, Guizhou, People’s Republic of China; 2 Pusat Kanser Tun Abdullah Ahmad Badawi, Universiti Sains Malaysia, Kepala Batas, Penang 13200 Malaysia; 3 Faculty Science Social and Humanities, School of Human Resource Development and Psychology, Universiti Teknologi Malaysia, Johor Bahru, Malaysia; 4 Department of Psychiatry and Mental Health, Faculty of Medicine, Universiti Sultan Zainal Abidin, Kuala Terengganu, Malaysia; 5 Women Cancer Unit Clinical Section of Advanced Medical and Dental Institute, Universiti Sains Malaysia, Bertam, Penang Malaysia; 6 The Second Affiliated Hospital of Xinxiang Medical University, Henan, People’s Republic of China; 7 Breast Cancer Translational Research Program, Universiti Sains Malaysia, Kepala Batas, Penang, Malaysia

**Keywords:** Acceptance and commitment therapy, Randomized controlled trial, Breast cancer, Helplessness, Quality of life

## Abstract

**Background:**

Breast cancer imposes substantial psychosocial challenges, with helplessness being a key determinant of reduced quality of life (QoL). Acceptance and Commitment Therapy (ACT) has shown promise in improving psychological flexibility, but its role in targeting helplessness and enhancing QoL in breast cancer patients has not been fully established. This randomized controlled trial (RCT) examined the effects of ACT on helplessness, QoL, and their domains.

**Methods:**

This two-arm, parallel, single-blind RCT recruited 80 breast cancer patients from a tertiary care center and randomly assigned them to either ACT (*n* = 40) or treatment-as-usual (TAU, *n* = 40). Participants completed the Malay Illness Cognition Questionnaire (ICQ-M) and the Functional Assessment of Cancer Therapy (General and Breast Cancer modules) at baseline (T1), post-intervention at 4 weeks (T2), and follow-up at 12 weeks (T3). ACT was delivered in four weekly sessions between T1 and T2. Data were analyzed using repeated-measures analysis of variance (ANOVA).

**Results:**

Sixty-six participants completed all assessments (ACT, *n* = 32; TAU, *n* = 34). Compared with TAU, ACT significantly reduced helplessness and improved overall QoL and all QoL domains across time points (all *p* < 0.05). Effects were maintained at 12-week follow-up.

**Conclusion:**

ACT is an effective psychosocial intervention for breast cancer patients, producing sustained improvements in QoL partly by reducing helplessness. Integrating ACT into supportive and palliative care may address critical psychological mechanisms affecting patient well-being.

**Trial registration:**

ClinicalTrials.gov, NCT05327153. Registered on April 6, 2022.

## Introduction

Breast cancer is now the second most common cancer worldwide, and the most common cancer in women. In 2022, 2.3 million new cases of breast cancer were reported globally, with 666,100 deaths [1]. Despite improved long-term survival rates, the quality of life (QoL) of breast cancer patients remains poor [2]. A considerable number of breast cancer patients encounter various simultaneous psychological symptoms throughout their cancer care journey, including distress, anxiety, and stressful situations, which could increase the risk of developing depression, cognitive impairment, and issues related to body image and sexual function [3]. These psychosocial burdens underscore the need to identify psychological factors that influence QoL and to develop interventions that address them effectively.

Cancer patients may experience helplessness due to the unpredictable course of their disease, invasive treatments, and prolonged uncertainty. 16% of cancer patients reported feeling weak and helpless several times a day or week, 49% occasionally, and only 35% reported not having this experience. However, among patients who reported “never feeling weak” in quantitative assessments, some still described helplessness situations related to cancer progression in open-ended responses [4]. For cancer patients, helplessness exerts severe multidimensional adverse impacts: far from being a pure psychological experience, it can trigger a series of physical symptoms including fatigue, low energy, sleep disturbances, gastrointestinal problems, and chronic pain [5], which in turn exacerbate emotional distress, cause persistent functional impairment in daily life, and lead to a significant decline in health-related QoL—a key outcome for cancer patients that is comprehensively assessed across physical, psychological, environmental, and social domains, as well as functional well-being [6]. The mind–body connection highlights that emotional distress can manifest physically, and, conversely, persistent physical symptoms can reinforce feelings of helplessness [7]. Therefore, psychological interventions targeting helplessness may help improve psychological well-being and reduce the burden of physical symptoms.

ACT has gained popularity due to its effectiveness across diverse clinical populations, including individuals with chronic illnesses such as cancer. For breast cancer patients, helplessness arises from a multifactorial cognitive-behavioral mechanism: unpredictable disease progression, invasive and adverse-effect-prone treatments, and prolonged prognostic uncertainty trigger negative cognitive appraisals and a loss of perceived control, which in turn lead to behavioral avoidance of disease-related distress and the persistence of helplessness [4]. The six core components of ACT include acceptance, cognitive defusion, contact with the present moment through mindfulness, self-as-context, value clarification, and committed action. These processes directly target the key cognitive-behavioral mechanisms underlying helplessness in breast cancer patients. The intervention encourages patients to act according to their identified value of life and accept their internal experiences, such as unpleasant thoughts or feelings, without passing judgment [8]. Specifically, cognitive defusion helps patients detach from helplessness-driven negative appraisals, reducing their emotional impact. Acceptance replaces behavioral avoidance with open engagement with distressing experiences, thereby breaking the “avoidance–helplessness” cycle. Mindful present-moment contact shifts focus from ruminating on uncertain outcomes to controlling behaviors. Finally, value clarification and committed action restore perceived control by guiding meaningful, value-consistent actions. Together, these strategies disrupt the formation and persistence of helplessness. ACT may assist breast cancer patients in addressing specific challenges and the psychological impacts of the illness, including psychological flexibility [9], coping with uncertainty [10], anxiety and depression [11], enhanced QoL [12], resilience [12], and posttraumatic growth [13]. Therefore, ACT is a viable treatment option for this population.

Existing research on helplessness interventions has been conducted across the general population, cancer patients, and breast cancer patients. For the general population, cognitive-behavioral interventions have demonstrated preliminary efficacy in reducing helplessness by modifying negative cognitive patterns [14]. For patients with cancer, several studies have begun to explore interventions specifically targeting helplessness. One pilot study demonstrated that an internet-based cognitive behavioral therapy (CBT) program reduced helplessness and achieved clinically significant improvements in outcomes for early-stage cancer patients [15]. For breast cancer patients, who face unique disease-related stressors, an RCT on a helplessness-specific intervention investigated an interactive self-help CBT workbook for newly diagnosed patients, demonstrating that CBT significantly reduced helplessness [16]. Notably, one study has investigated the potential of ACT for breast cancer patients. This quasi-experimental study, which focused on group-based ACT for chemotherapy recipients, showed that ACT significantly improved illness cognition and QoL [17]. These findings provide critical preliminary evidence for ACT’s efficacy in the breast cancer population.

Although ACT has several applications in managing cancer patients, data are still lacking on whether ACT profoundly affects helplessness and QoL among breast cancer patients. Hence, this study filled the research gap by evaluating the effect of ACT in reducing helplessness and improving QoL in breast cancer patients at three time points (baseline, post-intervention, and follow-up) and comparing it with treatment as usual (TAU).

## Methods

### Trial design and participants

This study is a two-arm, parallel-group, single-blind RCT comparing ACT with TAU. The study protocol was approved by the Human Research Ethics Committee of Universiti Sains Malaysia (USM) (Code: USM/JEPeM/22080569) and was conducted in accordance with the ethical standards of the 1964 Helsinki Declaration and its later amendments. The reporting of this study adheres to the CONSORT 2010 guidelines.

The sample size was determined through a priori power analysis using G*Power 3.1.9.2. Based on Han, et al. [17], who reported an effect size of 0.61 for ACT’s effect on disease acceptance in cancer patients, this effect size was adopted. With a two-tailed type I error of 0.05 and a desired power of 0.95, a total of 64 participants (32 per group) were required. This number was inflated by 20% to account for an anticipated dropout rate, resulting in a target recruitment of 80 participants.

Participants were recruited from the oncology outpatient clinics and inpatient wards of the Advanced Medical and Dental Institute (AMDI). Recruitment was carried out by a trained assistant who remained blinded to both the research hypotheses and the randomization sequence. This ensured that the presentation of study details, including risks and benefits, was conducted without biased influence or knowledge of participant allocation. The AMDI is a tertiary referral center for oncology patients in the northern region of Peninsular Malaysia. Before enrolling in the study, the participants provided informed consent, underscoring their voluntary engagement in the research. All participants provided written informed consent before enrollment and retained the right to withdraw from the study at any time.

Eligibility was assessed based on the following criteria. Inclusion criteria were: (1) histopathologically confirmed diagnosis of breast cancer; (2) currently receiving active anti-cancer treatment and not at the end-of-life stage (i.e., life expectancy ≥ 6 months, as assessed by the attending oncologist); (3) aged ≥ 18 years; and (4) willing to participate in the intervention and follow-up assessments. Patients were excluded if they: (1) had cognitive impairment (Mini-Mental State Examination score ≤ 24); (2) had psychiatric comorbidities (e.g., psychosis, bipolar disorder, posttraumatic stress disorder, substance or alcohol use) that would hinder study participation; or (3) were currently receiving or had previously received any other form of psychological therapy. Eligible participants were then invited to take part in the study.

### Participant randomization

Participants were randomized into two groups (ACT and TAU) using permuted block randomization with a 1:1 allocation ratio and a fixed block size of 4. The randomization sequence was computer-generated by a research assistant who was not involved in participant recruitment or intervention delivery. The allocation sequence was concealed in sequentially numbered, opaque, sealed envelopes prepared by an independent biostatistician not involved in any other aspect of the study. Each envelope contained the group assignment for a single participant and was opened only at the time of intervention allocation.

### Blinding

Due to the nature of the non‑pharmacological intervention, participant blinding was not feasible. This was clearly disclosed during informed consent, where all participants were informed of the randomization design. Research assistants facilitating recruitment, assessment, and analysis were naïve to randomization or intervention delivery, ensuring they were unaware of group allocation throughout the study. As a compensatory measure, control‑group participants were offered delayed ACT sessions free of charge after all follow‑up assessments were completed, ensuring benefit to all participants and addressing ethical concerns regarding unequal access to the intervention.

### Intervention

All ACT sessions were conducted in the daycare cancer outpatient department of the AMDI, consisting of four structured 60-minute individual sessions delivered weekly on a one-on-one basis. The intervention was designed to establish a therapeutic relationship and empower breast cancer patients to manage illness-related experiences. Session 1 involved therapist introductions and initial intake interviews to understand patients’ thoughts and feelings about their condition. Session 2 focused on accepting negative experiences and thoughts via creative hopelessness and metaphorical techniques to foster psychological acceptance and cognitive defusion. Session 3 introduced mindfulness exercises to enhance awareness of the “observing self” and alleviate fear and worry about the future. Session 4 guided patients to identify personal values and actionable goals, building confidence and proactive life engagement through tailored activities. Overall, the sessions aimed to support patients throughout their breast cancer journey and improve their QoL. Given the predominantly Malay-Muslim sample, specific cultural and religious adaptations were made to ensure the intervention’s relevance and acceptability: mindfulness exercises in Session 3 were integrated with Muslim pre-prayer ablution mindfulness rituals to align with religious practices, Session 4’s value clarification centered on core Malay-Muslim values such as family harmony and communal connection, intervention scheduling avoided Muslim religious activities. All these adaptations were reviewed by local Malay-Muslim mental health professionals to preserve the core therapeutic principles of ACT while fitting the cultural and religious context of the study sample.

All ACT interventions were delivered by a single licensed clinical psychologist with seven years of clinical experience and formal specialized training in ACT. A summary of the structured ACT sessions is provided in Table 1.

**Table 1:** Summary of ACT sessions

Session	Element in hexaflex	Activities	Duration
1. Introductory	The therapeutic relationship, initial intake interview, and introduction to ACT	Therapists introduce themselves and their professional backgrounds. Conduct an initial intake interview. Develop a case formulation	1 h
2. Show up	Contact with the present moment and self as context	Introduce Acceptance-creative hopelessness. Develop an alternative approach to negative experiences. Use metaphorical techniques.	1 h
3. Let it go	Acceptance and defusion	Explain “Self-as-context” as the observing self. Guide patients through mindfulness exercises. Use metaphorical exercises.	1 h
4. Get moving	Values and committed action	Help patients determine values and goals. Provide objective data on breast cancer diagnosis and treatment progress.	1 h

### Treatment fidelity

The therapists received ACT training from a psychiatrist and a clinical psychologist. To assess treatment fidelity, 15% of ACT session audio recordings were randomly selected via a computer-generated random number sequence (stratified by therapist and session 1–4 for representative sampling). These were evaluated by a psychiatrist and a clinical psychologist using the ACT Adherence Scale and ACT Competence Rating Scale; interrater reliability was 0.75 (correlation coefficient). Additionally, therapists consulted an experienced psychiatrist to resolve intervention-related difficulties during session delivery.

### Procedures

Following screening, participants were randomly assigned to either the intervention group or the TAU control group. At baseline (T1), they were administered the ICQ-M to assess their degree of helplessness in living with breast cancer and the Malay version of the Functional Assessment of Cancer Therapy–Breast (FACT-B) to assess QoL, including both general and breast cancer-specific domains. These instruments were administered again for the following assessment at T2 (immediately upon intervention completion at week 4) and T3 (12 weeks following intervention completion).

### Outcomes

#### Primary outcome

Helplessness was assessed via the ICQ-M. The original English version of the ICQ consists of three domains: helplessness, acceptance, and perceived benefits. In this scale, higher scores indicate more positive cognitive adaptation in each respective domain [18]. The ICQ-M was adapted and validated for the Malaysian cancer population and demonstrated good psychometric properties, with Cronbach’s α values ranging from 0.742 to 0.927. Factor analysis supported a two-factor structure, combining acceptance and perceived benefit into one domain and retaining helplessness as a separate domain [19]. To ensure consistency in interpretation, the helplessness domain was reverse-scored so that higher scores on all subscales reflected more adaptive illness cognition.

#### Secondary outcome

The perceived QoL among the breast cancer participants in this study was assessed by the Malay versions of the FACT-B. The FACT-B consists of 37 items and five domains (physical condition, social and family situation, emotional state, functional condition, and a breast cancer-specific module) [20]. The Malay FACT-B exhibited high internal consistency (Cronbach’s α = 0.88) and was adapted and validated for use in the breast cancer population in Malaysia [21].

### Statistical analysis

Data analysis was conducted using the Statistical Package for the Social Sciences (SPSS version 29). The participants’ sociodemographic and clinical characteristics are presented as frequencies and percentages. Independent t tests and chi-square tests were used to evaluate differences in continuous and categorical variables, respectively, between the ACT and TAU control groups. To examine differences between groups and across time points, a mixed-design analysis of variance (ANOVA) was conducted, with group as the between-subjects factor and time (T1, T2, T3) as the within-subjects factor. The Bonferroni post hoc correction was applied when there was a significant group × time interaction. The results are reported as the mean difference (95% confidence interval) and standard error. Effect sizes were calculated to assess changes in patients’ perceptions of measured variables with and without the ACT intervention. Partial eta squared was used as the preferred measure for evaluating effect size in ANOVA-like tests, as it accounts for the proportion of total variance attributable to the treatment effect while controlling for other variables.

## Results

### Sample characteristics

**Fig. 1:**
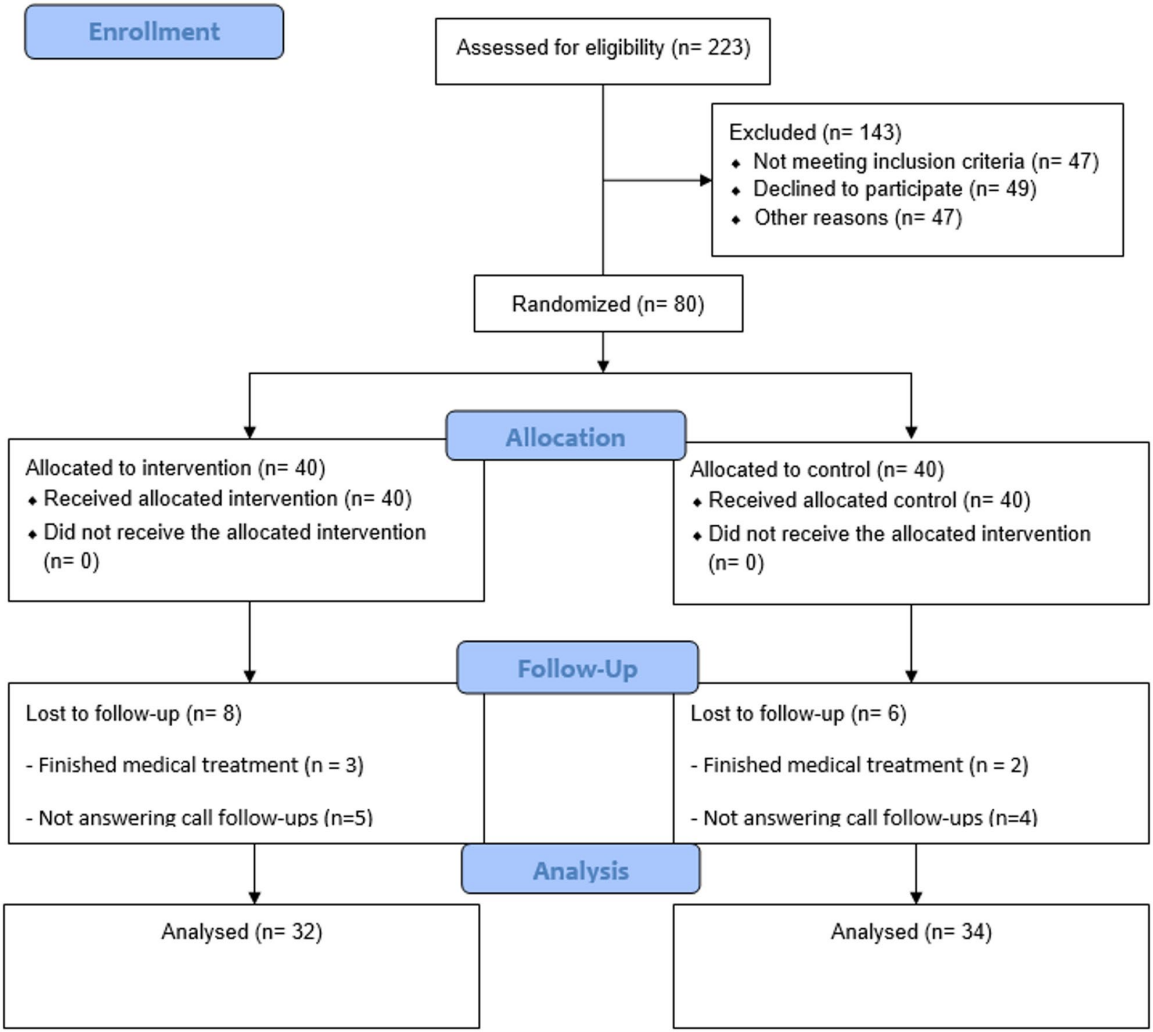
Trial flow diagram of study enrolment, allocation, and follow-up

Figure 1 summarizes the study’s recruitment, enrollment, allocation, follow-up, and analysis processes. Initially, 250 patients were approached, of whom 100 were excluded due to lack of interest (*n* = 55), transportation issues (*n* = 25), or other reasons (*n* = 20). The remaining 150 patients were screened for eligibility; of these, 50 did not meet the inclusion criteria, and 20 declined to participate due to time constraints, physical discomfort or fatigue, or unwillingness to communicate. Thus, 80 participants were enrolled and randomly assigned to the ACT or TAU group in a 1:1 ratio. Some participants were lost to follow-up or withdrew during the study, resulting in a final sample of 32 participants in the ACT group and 34 in the TAU group. Dropouts were unlikely to contribute to bias in the treatment effect analysis, as the number of dropouts was small, with ≤ 5% of total participants [22]. Additionally, all the ACT group participants received all four ACT sessions except for 2 participants (5%). No adverse effects or harm were reported during the intervention.

Table 2 presents the sociodemographic and clinical characteristics of participants in the ACT and TAU groups, with no significant difference observed between the two groups at baseline. Most participants were aged between 46 and 65 years. The sample was predominantly Malay and Muslim. Most participants were married, completed secondary or tertiary education, and reported a monthly household income below RM 4,500. The majority of participants had stage II–IV breast cancer. Treatment modalities varied, with chemotherapy and hormone therapy being the most common.

**Table 2:** Patient sociodemographic and clinical characteristics in the ACT and TAU control groups at baseline

Variables	*N* (%)		χ2	*p*
	ACT (*n* = 40)	Control (*n* = 40)		
Age (years)				
18–25	1(2.5)	0	1.531(3)	0.675
26–45	12(30.0)	13(32.5)		
46–65	23(57.5)	21(52.5)		
> 65	4(10.0)	6(15.0)		
Ethnicity				
Malays	36(90)	37(92.5)	0.214 (2)	0.899
Chinese	3(7.5)	2(5.0)		
Indians	1(2.5)	1(2.5)		
Religion				
Islam	37(92.5)	37(92.5)	1.333 (3)	0.721
Buddhism	2(5.0)	1(2.5)		
Hindu	1(2.5)	1(2.5)		
Christian	0	1(2.5)		
Monthly household income				
< RM 4,500	26(65)	27(67.5)	0.059 (2)	0.971
RM 4500-RM 11,000	13(32.5)	12(30)		
> RM 11,000	1(2.5)	1(2.5)		
Marital status				
Married	31(77.5)	30(75.0)	0.069 (1)	0.793
Single/divorcee/widow/widower	9(22.5)	10(25.0)		
Education level				
Primary	1(2.5)	4(10.0)	2.031 (2)	0.362
Secondary	21(52.5)	18(45.0)		
Tertiary	18(45.0)	18(45.0)		
Stage of cancer				
I	2(5.0)	2(5.0)	1.333 (3)	0.721
II	14(35.0)	14(35.0)		
III	14(35.0)	10(25.0)		
IV	10(25.0)	14(35.0)		
Current treatment				
Chemotherapy				
1st cycle	8(20.0)	5(12.5)	0.344 (5)	0.501
2nd cycle	7(17.5)	6(15.0)		
3rd cycle	2(5.0)	5(12.5)		
4th cycle	7(17.5)	6(15.0)		
Hormone Therapy	11(27.5)	16(40.0)		
Radiation therapy	5(12.5)	2(5.0)		

χ2: chi-square value; statistical significance: *p* < 0.05

### Primary outcome findings

At baseline, there were no statistically significant differences between the ACT and control groups in overall illness cognition, helplessness, acceptance, or perceived benefits (see Table 3). This indicates that the groups were comparable at baseline, thereby enhancing internal validity and minimizing potential confounding. However, significant group differences in illness cognition and helplessness emerged at T2 and T3. Compared with the control group, patients who received the 4-week ACT intervention showed greater improvements in illness cognition and a greater reduction in helplessness.

**Table 3:** Comparison of ICQ across the three time points between ACT and control group

Variable	Time-point	Mean (SD)		*p*
		ACT	Control	
Illness cognition	T1	52.63(9.34)	56.85(7.95)	0.051
	T2	60.47(8.26)	56.59(6.71)	0.040*
	T3	61.81(7.88)	56.97(7.00)	0.010*
Helplessness	T1	16.16(3.81)	17.00(4.19)	0.400
	T2	19.44(3.76)	16.79(4.19)	0.009**
	T3	20.47(2.87)	17.59(3.76)	< 0.001***
Acceptance & perceived benefits	T1	36.47(7.55)	39.85(6.69)	0.058
	T2	41.03(6.42)	39.79(5.37)	0.400
	T3	41.34(6.09)	39.38(5.55)	0.180

*p < 0.05, **p < 0.01, ***p < 0.001

Table 4 presents the mixed-model ANOVA results for illness cognition, helplessness, and acceptance and perceived benefits. Significant Group × Time interaction effects were found for all three variables (all *p* < 0.001), with partial eta squared (ηₚ²) values of 0.40, 0.27, and 0.23, respectively. A significant main effect of Time was observed for each variable, but no significant main effect of Group was detected. Bonferroni-corrected post hoc comparisons revealed significant improvements in the ACT group across multiple time points. For illness cognition, significant improvements were observed from T1 to T2 and T1 to T3. For helplessness, significant reductions were found across all time points (T1 to T2, T2 to T3, and T1 to T3). For acceptance and perceived benefits, a significant improvement was observed from T1 to T2. In contrast, the control group showed no significant changes across any time point for any of the variables.

**Table 4:** Mixed-model analysis variance of illness cognition, helplessness, acceptance and perceived benefits

Variable		F	*p*	ηp²	Significant Contrasts (Bonferroni)
Illness cognition	Group x Time	43.23	< 0.001	0.40	ACT: T1 < T2, T1 < T3 Control: NS
	Group	0.66	0.419	0.01	
	Time	42.67	0.001	0.40	
Helplessness	Group x Time	23.25	< 0.001	0.27	ACT: T1 < T2, T1 < T3, T2 < T3 Control: NS
	Group	3.24	0.076	0.05	
	Time	32.81	< 0.001	0.34	
Acceptance & perceived benefits	Group x Time	18.71	< 0.001	0.23	ACT: T1 < T2 Control: NS
	Group	0.002	0.966	0.00	
	Time	14.72	< 0.001	0.19	

*NS* not significant, *T1* baseline, *T2* post-intervention, *T3* 12-week follow-up

To further clarify the between-group effects of the ACT intervention on each dimension at different time points, Table 5 presents the mean differences in scores, standard errors, and corresponding p-values for the ACT group compared to the control group across three dimensions at T1, T2, and T3. The results revealed a statistically significant mean difference in the Illness Cognition dimension at T2 and T3, with a marginally significant difference at T1. For the Helplessness dimension, no significant between-group difference was observed at T1, whereas significant differences emerged at T2 and T3. Regarding the acceptance and perceived benefits dimension, no statistically significant differences between-group mean differences were found at any of the three time points, with only a marginal trend toward significance at T1.

**Table 5:** Between-subject simple effect analyses of illness cognition, helplessness, and acceptance & perceived benefits (ACT Group vs. Control Group) at each time point

Variable	Time point	Mean difference of ACT compared to control groups (95% CI)	Standard error	*p*-value
Illness cognition	T1	-4.228(-8.483 to 0.027)	2.130	0.051
	T2	3.881(0.191 to 7.570)	1.847	0.040
	T3	4.842(1.182 to 8.502)	1.832	0.010
Helplessness	T1	-0.844(-2.818 to 1.130)	0.988	0.396
	T2	2.643 (0.682 to 4.605)	0.982	0.009
	T3	2.881 (1.228 to 4.533)	0.827	< 0.001
Acceptance & perceived benefits	T1	-3.384(-6.887 to 0.119)	1.754	0.058
	T2	1.237(-1.666 to 4.141)	1.453	0.398
	T3	1.961(-0.901 to 4.823)	1.433	0.176

### Secondary outcome findings

At baseline, no significant group differences were observed for overall QoL or any of its domains, including physical, social/family, emotional, and functional well-being, or the breast cancer-specific subscale (see Table 6). Compared with the control group, patients who received the ACT intervention reported significantly higher QoL scores at T2 across all domains except social/family well-being. These improvements were sustained at T3.

**Table 6:** Comparison of QoL over three time points between the ACT group and the control group

Variables	Time-point	Mean (SD)		*p*
		ACT	Control	
Overall QoL	T1	99.67 (21.18)	104.46 (20.64)	0.356
	T2	115.60 (17.07)	101.51 (20.59)	0.004**
	T3	118.99 (15.82)	100.77 (21.10)	< 0.001***
Physical	T1	18.28 (6.85)	18.53 (7.00)	0.885
	T2	21.16 (5.36)	17.62 (7.50)	0.032*
	T3	21.50 (4.30)	17.21 (7.34)	0.005**
Family/Social	T1	20.45 (4.25)	22.49 (4.23)	0.056
	T2	23.57 (3.21)	22.69 (4.24)	0.880
	T3	24.11 (2.62)	23.06 (4.24)	0.234
Emotional	T1	17.31 (5.03)	18.21 (4.87)	0.466
	T2	20.19 (3.33)	18.12 (4.21)	0.031*
	T3	20.88 (2.52)	17.41 (4.38)	< 0.001***
Functional	T1	18.69 (6.49)	20.38 (6.94)	0.310
	T2	22.72 (4.78)	19.12 (6.15)	0.010*
	T3	22.63 (4.26)	19.00 (6.18)	0.008**
Breast Cancer Subscale	T1	25.03 (6.02)	24.85 (6.06)	0.180
	T2	27.97 (5.22)	23.97 (6.57)	0.008**
	T3	29.88 (6.04)	24.09 (6.69)	< 0.001***

*p < 0.05, **p < 0.01, ***p < 0.001

Table 7 displays the mixed-model analysis of variance results for overall QoL and its subdimensions as well as the Breast Cancer Subscale in breast cancer patients, showing significant Time × Group interaction effects for all QoL variables with ηₚ² values ranging from 0.15 to 0.41. A significant main effect of Time was observed for all dimensions, while the main effect of Group was only significant for overall QoL and the Breast Cancer Subscale, with no significant Group main effect for the other subdimensions. Bonferroni-corrected significant contrasts indicated the ACT group had progressive, continuous improvements in overall QoL and the Breast Cancer Subscale, along with significant improvements from T1 to T2 and T1 to T3 in Physical, Family/Social, Emotional and Functional well-being; in contrast, the control group exhibited no significant changes across all time points for any QoL variable.

**Table 7:** Mixed-model analysis of variance of QoL in breast cancer patients

Variables		F	*p*	ηp²	Significant Contrasts (Bonferroni)
Overall QoL	Time x Group	43.79	< 0.001	0.41	ACT: T1 < T2< T3 Control: NS
	Time	20.37	< 0.001	0.24	
	Group	4.02	0.049	0.06	
Physical	Time x Group	12.95	< 0.001	0.17	ACT: T1 < T2, T1 < T3 Control: NS
	Time	2.71	0.070	0.04	
	Group	2.81	0.099	0.04	
Family/Social	Time x Group	11.33	< 0.001	0.15	ACT: T1 < T2, T1 < T3 Control: NS
	Time	18.71	< 0.001	0.23	
	Group	0.002	0.967	< 0.001	
Emotional	Time x Group	23.19	< 0.001	0.27	ACT: T1 < T2, T1 < T3 Control: NS
	Time	9.37	< 0.001	0.13	
	Group	2.81	0.098	0.04	
Functional	Time x Group	30.37	< 0.001	0.32	ACT: T1 < T2, T1 < T3 Control: NS
	Time	7.67	0.001	0.11	
	Group	1.79	0.186	0.03	
Breast Cancer Subscale	Time x Group	17.14	< 0.001	0.21	ACT: T1 < T2, T1 < T3, T2 < T3 Control: NS
	Time	8.69	< 0.001	0.12	
	Group	5.63	0.021	0.08	

*NS* not significant, *T1* baseline, *T2* postintervention, *T3* 12-week follow-up

For the overall QoL total score, the ACT group showed a progressive, significant improvement (T1-T2: 15.930, T1-T3: 19.316), with these increases far exceeding the FACT-B minimum clinically important difference (MCID) (7–8) [23] and corresponding to a large clinical effect size (ηₚ²=0.41, *p* < 0.001). This confirms the large statistical effect translates to a patient-perceptible, clinically meaningful QoL improvement, which was specific to the ACT intervention and produced a significant intergroup advantage at T2/T3.

Table 8 presents the between-group mean differences, 95% confidence intervals, and statistical significance of QoL outcomes between the Acceptance and Commitment Therapy group and the control group across three time points for overall QoL and five specific domains. At T1, there were no significant differences between the two groups in any of the measured outcomes. Following the intervention, the ACT group showed significant improvements compared to the control group in overall QoL, physical, emotional, functional, and breast-cancer-specific well-being, apart from the family/social domain. These significant benefits were sustained or further enhanced at T3, particularly in overall QoL, physical, emotional, functional, and breast cancer specific well-being, where the ACT group continued to demonstrate highly significant advantages. The results visually illustrate the clear and progressive positive effect of the ACT intervention on improving QoL in breast cancer patients over time, with evidence of effect maintenance even after the intervention concluded.

**Table 8:** Comparison of ACT vs. control group on qol domains across three time points

Variable	Time point	Mean difference of ACT compared to control groups (95% CI)	Standard error	*p*-value
Overall QoL	T1	-4.787(-15.072 to 5.498)	5.148	0.356
	T2	14.087(-5.498 to 15.072)	4.672	0.004
	T3	18.218(9.003 to 27.434)	4.613	< 0.001
Physical	T1	-0.248(-3.654 to 3.158)	1.705	0.885
	T2	3.539(0.316 to 6.762)	1.613	0.032
	T3	4.294(1.310 to 7.278)	1.494	0.005
Family/Social	T1	1.049(-0.695 to 2.793)	1.043	0.056
	T2	1.049(-0.695 to 2.793)	0.931	0.348
	T3	1.049(-0.695 to 2.793)	0.873	0.234
Emotional	T1	-0.893(-3.326 to 1.539)	1.218	0.466
	T2	2.070(0.196 to 3.944)	0.938	0.031
	T3	3.463(1.692 to 5.235)	0.887	< 0.001
Functional	T1	-1.695(-5.005 to 1.615)	1.657	0.310
	T2	3.601(0.880 to 6.323)	1.362	0.010
	T3	3.625(0.999 to 6.251)	1.315	0.008
Breast Cancer Subscale	T1	0.178(-2.793 to 3.149)	1.487	0.905
	T2	3.998(1.069 to 6.927)	1.466	0.008
	T3	5.787(2.646 to 8.928)	1.572	< 0.001

## Discussion

The present study demonstrated that ACT significantly reduced feelings of helplessness and enhanced QoL among breast cancer survivors, with these benefits sustained at the 12-week follow-up. Helplessness often arises from cognitive fusion with distressing thoughts such as “I can’t cope” or “there’s no way out.” ACT directly targets this cognitive process through cognitive defusion and the self-as-context perspective, enabling patients to step back from overwhelming internal experiences and regain psychological flexibility and a sense of agency. The pronounced reduction in helplessness may also be influenced by the clinical stage of diagnosis, as feelings of helplessness are often most intense early after diagnosis, when patients face emotional shock, uncertainty about prognosis, and loss of control [24]. In this study, most participants were diagnosed within the past year, making helplessness a particularly salient and responsive target for intervention.

This 4-session ACT intervention was structured to sequentially build psychological flexibility and mitigate helplessness via hexaflex-aligned core elements and tailored techniques: Session 1 established a therapeutic alliance, completed intake, and introduced core ACT concepts with a single sheet of paper metaphor to lay an early foundation for acceptance and defusion, preliminarily reducing cancer-related distress avoidance and helplessness; Session 2 (contact with the present moment/self as context) deepened this work through acceptance and creative hopelessness training, using a culturally adapted Chinese tangram metaphor to facilitate cognitive defusion, which directly alleviated emotional helplessness and improved emotional well-being by helping patients separate their self from overwhelming negative cognitions; Session 3 formalized the self-as-context concept and guided mindfulness exercises (e.g., breath-focused awareness, body scans), whose present-moment awareness mechanism reduced anxious rumination and somatic hypervigilance, thereby improving physical and emotional well-being by relieving cancer-related fatigue and emotional rumination; Session 4 guided patients to identify core personal values and translate them into concrete goals, bridging abstract ACT skills with real-world actions to counteract powerlessness—this values-aligned committed action helped patients reconnect with social roles and regain functional activity capacity, further reducing helplessness and boosting overall QoL [25].

Furthermore, the lack of sustained between-group difference in acceptance and perceived benefits is due to the time-intensive nature of illness acceptance, which requires longer support beyond the 4-session ACT intervention and 12-week follow-up. For family/social well-being, the null finding stems from baseline homogeneity of social support across both groups, as adequate support in the control group limited the intervention’s ability to create a measurable gap. Additionally, the absence of significant Group main effects for most variables reflects the time-dependent nature of ACT’s effects, which fosters gradual improvements rather than immediate baseline differences, aligning with ACT’s skill-building core.

Consistent with prior research, ACT promotes hope by enhancing present-moment awareness, clarifying personal values, and facilitating committed action toward meaningful goals [26, 27]. Increased hope, in turn, is associated with reduced helplessness and improved QoL [28–30]. Over time, patients may also experience gradual gains in perceived benefit and illness acceptance as they continue to apply ACT principles [31]. ACT in this study enhanced multiple QoL domains, including physical health, emotional well-being, social relationships, functionality, and breast cancer-specific QoL. The largest effect was observed in social relationships, likely due to increased psychological flexibility allowing patients to adopt more approach-oriented coping strategies, such as seeking emotional and instrumental support [32]. Given its transdiagnostic applicability, ACT may also improve physical health and functional outcomes by addressing comorbid medical and psychosocial conditions common among cancer survivors, including fatigue, chronic pain, and stigma [33, 34]. Collectively, these findings highlight ACT as a promising psychosocial intervention to enhance QoL within the broader framework of palliative care.

Several limitations should be acknowledged. First, the sociodemographic characteristics of the participants lack detailed clinical characteristics of the breast cancer patients. Second, no data on patient treatment adherence was collected, including key indicators such as attendance rates at ACT sessions and completion of post-session homework exercises. This omission precludes a formal analysis of the potential association between treatment adherence and intervention outcomes, which limits our ability to further elucidate the factors driving the observed therapeutic effects of the ACT intervention. Third, the study employed a treatment-as-usual control group, which means the observed benefits of the ACT intervention could partially be attributed to increased therapeutic attention from the one-on-one structured sessions, rather than the specific core components of ACT itself. Additionally, the present study only included a 12-week follow-up period, which restricts the assessment of the long-term sustainability of the ACT intervention’s benefits for breast cancer survivors.

Clinically, these results provide evidence that ACT can be integrated into supportive and palliative care programs for breast cancer patients. By targeting helplessness, ACT helps patients regain a sense of agency and hope, ultimately contributing to improved overall well-being. For clinical implementation, this brief, skill-based ACT program is well-suited for delivery by oncology mental health clinicians, oncology nurses, or trained psychosocial care providers within hospital-based cancer care centers or community palliative care services, as its 4-session format aligns with the time constraints of clinical practice and cancer patients’ treatment schedules. By targeting and mitigating helplessness through these structured, actionable ACT skills, clinical care teams can help breast cancer patients regain a sense of agency and hope, foster psychological flexibility, and ultimately improve their overall well-being alongside standard medical treatment. Future studies should explore additional psychosocial interventions aimed at reducing helplessness and confirm these findings in larger, more diverse samples using double-blinded RCT designs. Overall, this study underscores the importance of addressing helplessness as a mechanism for enhancing QoL among breast cancer survivors, with benefits sustained up to 12 weeks post-intervention.

## Data Availability

The data that support the findings of this study are available on request from the corresponding author (N.S.M).
